# *N*-Palmitoylethanolamide-Oxazoline Protects against Middle Cerebral Artery Occlusion Injury in Diabetic Rats by Regulating the SIRT1 Pathway

**DOI:** 10.3390/ijms20194845

**Published:** 2019-09-29

**Authors:** Roberta Fusco, Maria Scuto, Marika Cordaro, Ramona D’Amico, Enrico Gugliandolo, Rosalba Siracusa, Alessio Filippo Peritore, Rosalia Crupi, Daniela Impellizzeri, Salvatore Cuzzocrea, Rosanna Di Paola

**Affiliations:** 1 Department of Chemical, Biological, Pharmaceutical and Environmental Sciences, University of Messina, Viale Ferdinando Stagno D’Alcontres, n 31, 98166 Messina, Italy; rfusco@unime.it (R.F.); cordarom@unime.it (M.C.); rdamico@unime.it (R.D.); egugliandolo@unime.it (E.G.); rsiracusa@unime.it (R.S.); aperitore@unime.it (A.F.P.); rcrupi@unime.it (R.C.); dimpellizzeri@unime.it (D.I.); 2 Department of Biomedical and Biotechnological Sciences, University of Catania, 95131 Catania, Italy; mary-amir@hotmail.it; 3 Department of Pharmacological and Physiological Science, Saint Louis University School of Medicine, 1402 South Grand Blvd, St. Louis, MO 63104, USA

**Keywords:** ischemic stroke, PEA-OXA, inflammation

## Abstract

Diabetes causes various macrovascular and microvascular alterations, often culminating in major clinical complications (first of all, stroke) that lack an effective therapeutic intervention. *N*-palmitoylethanolamide-oxazoline (PEA-OXA) possesses anti-inflammatory and potent neuroprotective effects. Although recent studies have explained the neuroprotective properties of PEA-OXA, nothing is known about its effects in treating cerebral ischemia. Methods: Focal cerebral ischemia was induced by transient middle cerebral artery occlusion (MCAo) in the right hemisphere. Middle cerebral artery (MCA) occlusion was provided by introducing a 4–0 nylon monofilament (Ethilon; Johnson & Johnson, Somerville, NJ, USA) precoated with silicone via the external carotid artery into the internal carotid artery to occlude the MCA. Results: A neurological severity score and infarct volumes were carried out to assess the neuroprotective effects of PEA-OXA. Moreover, we observed PEA-OXA-mediated improvements in tissue histology shown by a reduction in lesion size and an improvement in apoptosis level (assessed by caspases, Bax, and Bcl-2 modulation and a TUNEL assay), which further supported the efficacy of PEA-OXA therapy. We also found that PEA-OXA treatment was able to reduce mast cell degranulation and reduce the MCAo-induced expression of NF-κB pathways, cytokines, and neurotrophic factors. Conclusions: based on these findings, we propose that PEA-OXA could be useful in decreasing the risk of impairment or improving function in ischemia/reperfusion brain injury-related disorders.

## 1. Introduction

Diabetes mellitus (DM) is a metabolic disorder connected with chronic hyperglycemia, which has been identified as enhancing systemic oxidative stress, inclining patients toward diabetic complications. World Health Organization (WHO) data show that around 386 million people in the world are now affected by diabetes, which is a main risk factor for atherosclerotic diseases, such as acute brain ischemia [1,2]. Additionally, it has been well demonstrated that diabetic patients have a higher possibility of stroke than nondiabetic patients do and are more likely to have a poor prognosis and increased mortality after stroke [3,4,5]. Cerebral ischemia is a consequence of an unexpected decrease or loss of blood passage to the brain area, which corresponds with a loss of neurological function. This disorder can lead to quadriplegia and difficulties with thinking, memory, and language. Often the restoration of blood flow to the ischemic area is one of the most useful strategies in clinical stroke treatment, but it can exacerbate neurocognitive deficits and give origin to additional damage. The early responders to this injury are the major immune system factors [6,7]. It has been well described that inflammation is an important characteristic of ischemic brain tissue [8,9]. This activation causes the infiltration of several inflammatory cell types and the production of proinflammatory mediators in the damaged tissue, free radical generation, oxidative stress, apoptosis, and/or axonal injury, ending in cellular degeneration, altered neural circuits, and impaired synaptic plasticity and transmission [10,11,12]. During hyperglycemia, NF-kB is quickly activated in vascular cells, developing in a subsequent intensification of leukocyte adhesion and the transcription of proinflammatory cytokines [13,14,15]. Recent studies, performed using experimental animal models, have helped to identify a wide selection of neuroprotective/anti-inflammatory compounds (e.g., inhibitors of inducible nitric oxide synthase and kinases, antiepileptics, polyphenols, and antioxidants) [16,17,18,19,20]. Often these molecules have shown few positive outcomes [21,22]. However, animal models offer the possibility to collect information to limit stroke severity, and they display translational potential in improving future stroke consequences [21,23]. The reason for the little efficacy of these approaches could be that they target only neuronal cell protection without a safeguard for cerebral blood vessels from secondary damage from inflammation and reactive oxygen/nitrogen species. Moreover, several anti-inflammatory strategies have been described that have displayed success in experimental animal models of stroke [24,25]; however, their translation into clinical trials has been disappointing [26,27]. In this paper, we would like to focus our attention on a class of lipid-signaling molecules, the *N*-acylethanolamines (NAEs) [28,29], which are produced in response to tissue stress and injury to prevent additional damage and restore homeostatic balance [30]. It is well known that one of these NAEs, *N*-palmitoylethanolamide (PEA), allows for the maintenance of cellular homeostasis, acting in the resolution of the inflammatory pathway [31,32]. In particular, PEA has shown neuroprotective and anti-neuroinflammatory effects [33,34,35,36]. This anti-inflammatory effect can also be gained by upregulating endogenous PEA levels and targeting its principal catabolic enzyme, *N*-acylethanolamine-hydrolyzing acid amidase (NAAA) [37], through the modulation of its degradation [37,38,39,40,41]. PEA on-demand production exerts an adjusting effect on glia and mast cells involved in neuroinflammation [31,32] and requests that PEA pleiotropic actions [31] be organized by a mechanism, allowing for inactivation. It has been proposed that NAAA can control PEA levels, preserving its on-demand synthesis and degradation [42]. Recent evidence has shown that pharmacological management of NAAA with the oxazoline of PEA (2-pentadecyl-2-oxazoline (PEA-OXA)) [43] had beneficial effects on secondary neuroinflammatory events associated with the spinal cord, traumatic brain injury, and Parkinson’s disease in mice [44,45]. Starting from this evidence, the aim of this study was to investigate the effects of PEA-OXA on secondary neuroinflammatory events induced by transient middle cerebral artery occlusion (MCAo) in diabetic rats.

## 2. Results

### 2.1. Effect of PEA-OXA Treatment on Regional Cerebral Blood Flow (rCBF) and Ischemic Brain Damage

**Figure 1:**
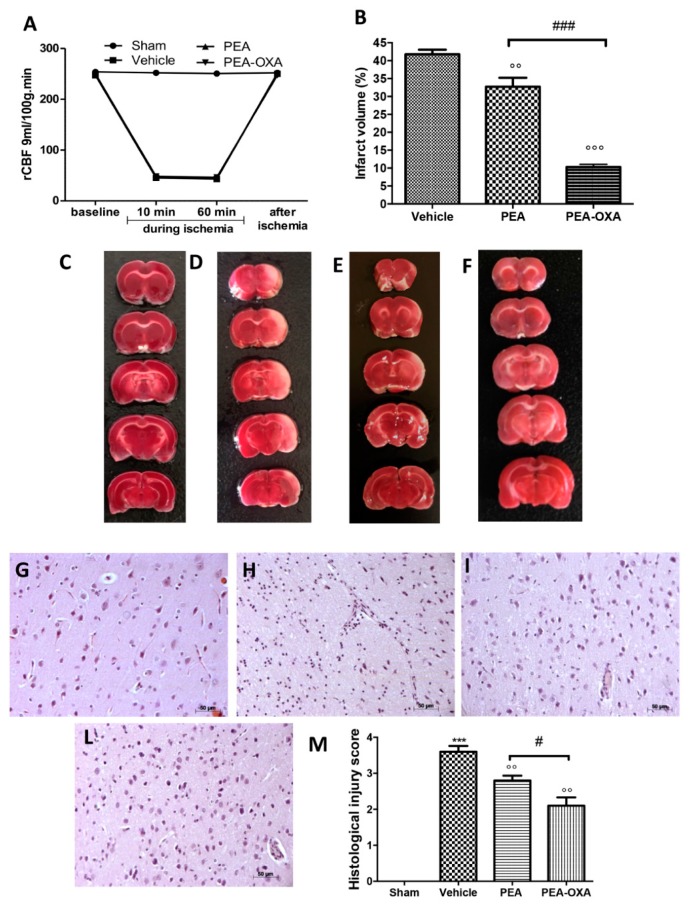
Efficacy of PEA-OXA on rCBF and the ischemic area induced by transient MCAo: rCBF was controlled to confirm the success of the induction of the transient ischemia. This was monitored at baseline (10 min before), at 10 and 60 min during ischemia, and 5 min after ischemia (**A**). TTC staining of brain sections was performed 24 h after MCAo. Tissues from vehicle-treated animals displayed the presence of an unstained area (**B**,**D**) compared to the sham group (**B**,**C**). PEA-OXA administration (**B**,**F**) reduced this infarcted area more effectively than did the PEA treatment (**B**,**E**). A histological analysis of brain samples from vehicle-treated rats revealed the loss of neurons (**H**,**M**) compared to the sham group (**G**,**M**), which was ameliorated by PEA administration (**I**,**M**). PEA-OXA administration displayed more protective effects compared to the PEA treatment (**L**,**M**). For the histology, a 20× magnification is shown (50-µm scale bar). A *p*-value of less than 0.05 was considered significant: °° *p* < 0.01 versus vehicle; *** *p* < 0.001 versus sham; °°° *p* < 0.001 versus vehicle; # *p* < 0.05 versus PEA; ### *p* < 0.001 versus PEA.

The monitoring of rCBF displayed successful MCAo (Figure 1A). At the same time points, no statistical difference was detected in rCBF between sham, PEA-, and PEA-OXA-administered animals. Additionally, 24 h after ischemia/reperfusion, the animals showed infarcts affecting the striatum and cortex (Figure 1B,D). The PEA-OXA-administered group (Figure 1B,F) had significantly less tissue damage 24 h after transient MCAo compared to sham-operated animals (Figure 1B,C) and PEA-treated animals (Figure 1B,E). A histological analysis displayed healthy cerebral neurons in the control group (Figure 1G,M), while (24 h after transient MCAo) vehicle-treated animals revealed a paucity of intact neurons (Figure 1H,M). In tissues obtained from PEA-administered animals, the corresponding area showed protection from neuronal cell loss (Figure 1I,M). This protection was significantly higher in the PEA-OXA-administered animals (Figure 1L,M).

### 2.2. Effect of PEA-OXA Treatment on MCAo-Induced Mast Cell Degranulation

**Figure 2:**
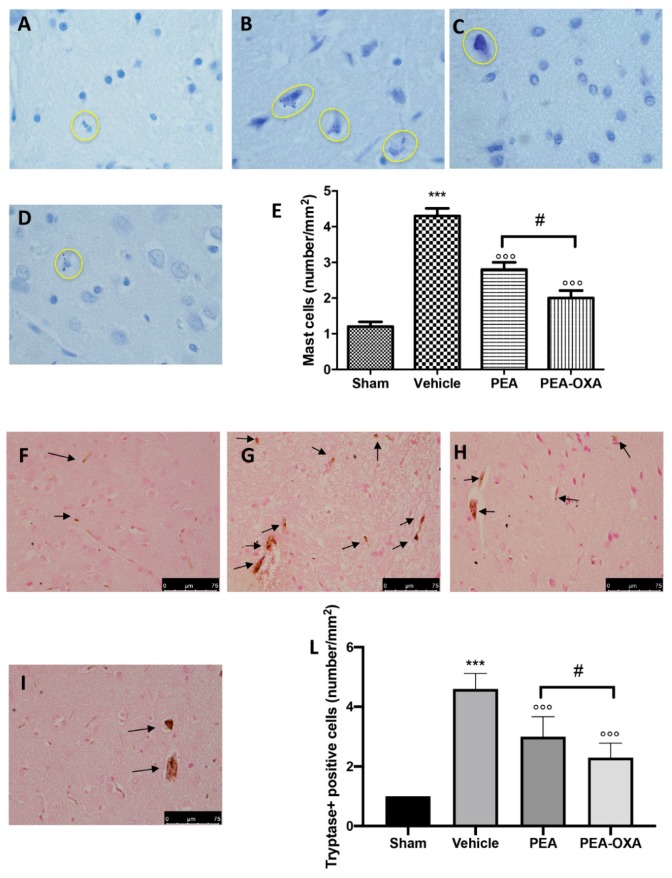
Efficacy of PEA-OXA on mast cell infiltration and degranulation induced by transient MCAo. An increased number of mast cells were detected in tissues from vehicle-treated animals (**B**,**E**) compared to the control (**A**,**E**). PEA and PEA-OXA-treated ischemic rats showed fewer cells of this type (**C**–**E**). An increased expression of tryptase (**G**,**L**) was found in sections obtained from vehicle-treated rats compared to the sham-operated animals (**F**,**L**). PEA-OXA administration reduced tryptase expression more effectively than did PEA (**H**,**I**,**L**). The number of mast cells was counted in three sections per animal and is presented as the number of positive cells per high-power field. For the mast cells, a 100× magnification is shown (10-µm scale bar). For the immunohistochemistry, a 40× magnification is shown (75-µm scale bar). A *p*-value of less than 0.05 was considered significant: *** *p* < 0.001 versus sham; °°° *p* < 0.001 versus vehicle; # *p* < 0.05 versus PEA.

Toluidine blue staining was performed to investigate mast cell infiltration and degranulation in the injured tissue. In brain sections from vehicle-treated animals, an increased number of mast cells were identified (Figure 2B,E) compared to the tissues from sham-operated rats (Figure 2A,E). In samples from animals administered PEA, fewer of these cells were observed (Figure 2C,E). In addition, tissues collected from PEA-OXA-administered animals revealed fewer mast cells than did tissues from vehicle-treated animals (Figure 2D,E). An immunohistochemical analysis of tryptase expression showed increased staining in tissue sections from vehicle-treated animals [46] (Figure 2G, see densitometric analysis in Figure 2L), which was reduced in samples from PEA-OXA-administered animals (Figure 2I, see densitometric analysis in Figure 2L). PEA administration was also able to reduce tryptase expression (Figure 2H, see densitometric analysis in Figure 2L), but PEA-OXA treatment was more effective. The basal staining of tryptase (Figure 2F, see densitometric analysis in Figure 2L) was detected in the sham-operated animals.

### 2.3. Effect of PEA-OXA Treatment on MCAo-Induced SIRT1 and UCP2 Expression and Redox Status 

**Figure 3:**
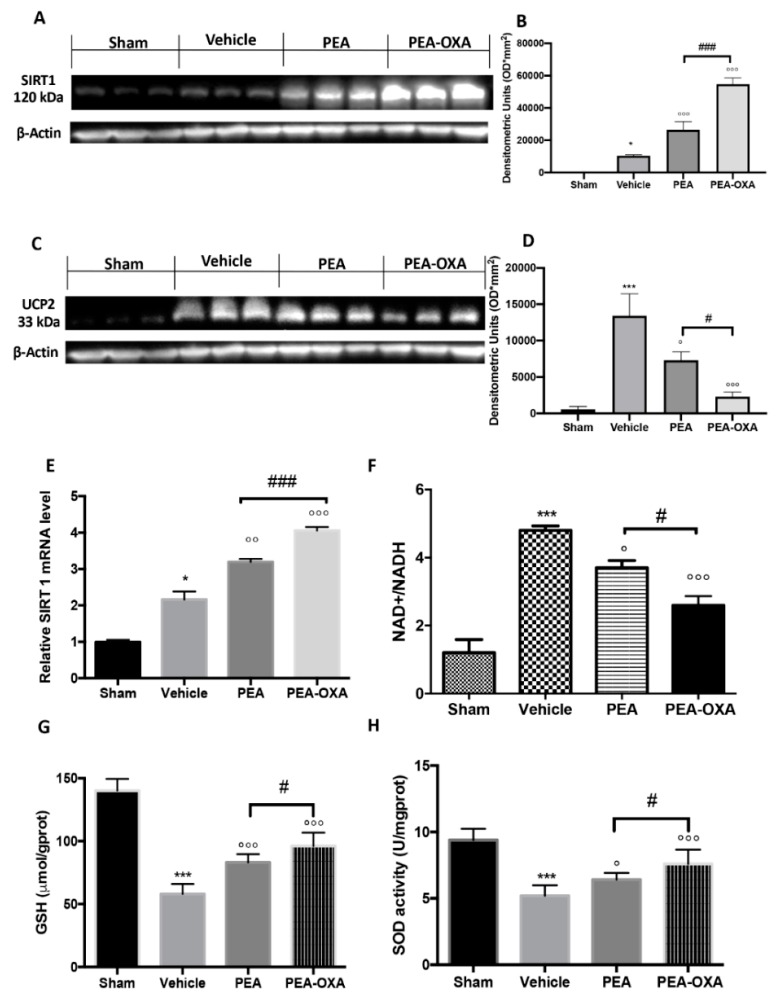
The efficacy of PEA-OXA on SIRT1 and UCP2 expression, the NAD^+^/NADH ratio, GSH, and SOD activity (induced by transient MCAo). A western blot analysis of SIRT1 showed basal expression that was significantly increased after MCAo. After PEA-OXA treatment, this expression was significantly upregulated, while after PEA administration, it increased less (**A**,**B**). Real-time PCR analysis of the *SIRT1* gene displayed the same trend (**E**). UCP2 expression in vehicle-treated animals was significantly increased compared to the sham-operated animals. PEA-OXA administration was able to significantly restore the basal levels (**C**,**D**). PEA displayed less protection. The NAD^+^/NADH ratio was reduced by PEA-OXA treatment compared to the vehicle-treated animals (**E**). MCAo reduced GSH (**G**) and SOD (**H**) activity in vehicle-treated animals, while PEA-OXA administration restored the basal levels. A *p*-value of less than 0.05 was considered significant: * *p* < 0.05 versus sham; ° *p* < 0.05 versus vehicle; °° *p* < 0.01 versus vehicle; *** *p* < 0.001 versus sham; °°° *p* < 0.001 versus vehicle; # *p* < 0.05 versus PEA; ### *p* < 0.001 versus PEA.

To understand the molecular mechanism by which PEA-OXA acts, we evaluated (by western blot) the modulation of SIRT1 (silent information regulator 1) and UCP2 (uncoupling protein 2) expression. PEA-OXA administration increased SIRT1 expression compared to the vehicle group (Figure 3A and the densitometric analysis in Figure 3B), while PEA treatment displayed less of an increase (Figure 3A and the densitometric analysis in Figure 3B). SIRT1 gene expression was increased in the PEA-OXA-administered animals compared to the vehicle group, while PEA-treated animals showed less upregulation (Figure 3E). Treatment with PEA-OXA also diminished UCP2 expression compared to the vehicle-treated rats (Figure 3C and the densitometric analysis in Figure 3D), while PEA treatment displayed less efficacy (Figure 3C and the densitometric analysis in Figure 3D). To observe the effect of PEA-OXA administration on redox status, the NAD+/NADH ratio (Figure 3F), the GSH level (Figure 3G), and SOD activity (Figure 3H) after MCAo were examined. The results showed that GSH and SOD were increased after PEA-OXA administration compared to the vehicle-treated animals, while PEA administration showed less efficacy (Figure 3G,H). Compared to the sham-treated animals, the NAD+/NADH ratio was higher in the vehicle-treated rats, while PEA-OXA treatment restored its levels (Figure 3F). PEA administration displayed less efficacy in the increase of NAD+/NADH (Figure 3F).

### 2.4. Effects of PEA-OXA Treatment on MCAo-Induced Apoptosis

**Figure 4:**
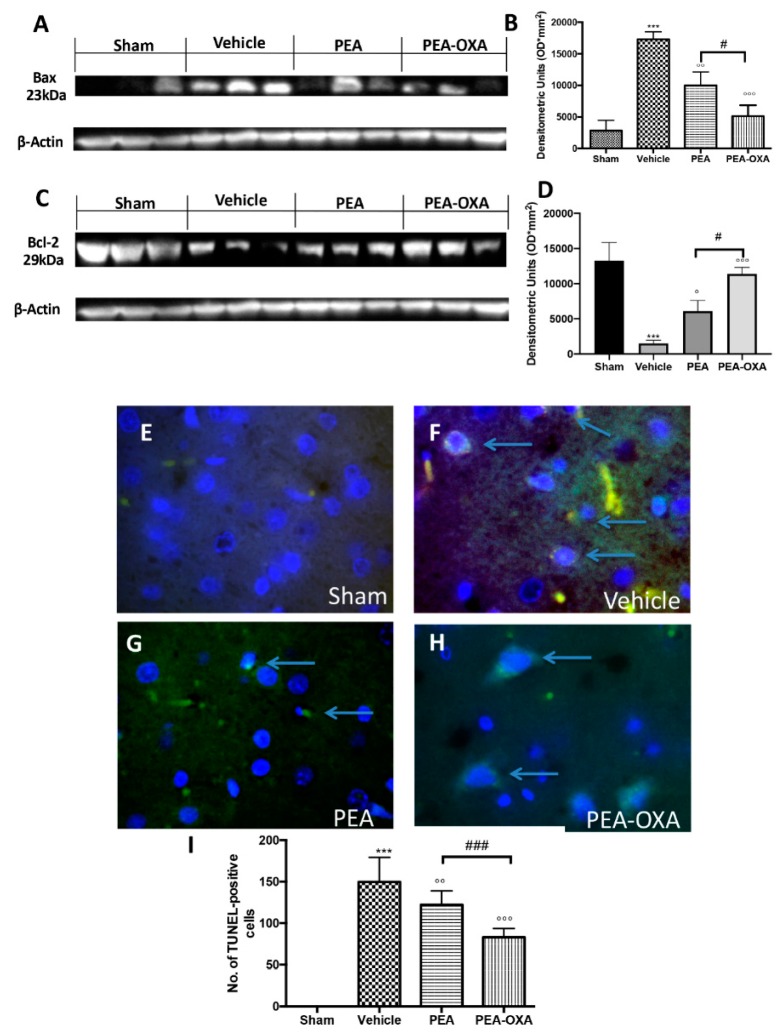
Efficacy of PEA-OXA on apoptosis induced by transient MCAo. In western blot analyses, a minimal expression of Bax in brain samples taken from sham rats was detected. PEA-OXA was able to decrease Bax expression in the MCAo-induced group (**A**,**B**). On the other hand, a basal level of Bcl-2 was present in brain tissue collected from sham rats, while the expression of Bcl-2 was significantly lower in the vehicle group. PEA-OXA treatment was able to increase the expression of Bcl-2 at levels similar to the sham group (**C**,**D**). Treatment with PEA at the same dose was less beneficial (**A**–**D**). A low level of TUNEL-positive staining was detected in the sham group (**E**,**I**). PEA-OXA (**H**,**I**) administration reduced the number of TUNEL-positive cells compared to the MCAo group (**F**,**I**). PEA treatment showed less efficacy (**G**,**I**). The number of TUNEL-positive cells was counted in three sections per animal and is presented as the number of positive cells per high-power field. For TUNEL staining, a 100× magnification is shown (10-µm scale bar). A *p*-value of less than 0.05 was considered significant: ° *p* < 0.05 versus vehicle; °° *p* < 0.01 versus vehicle; *** *p* < 0.001 versus sham; °°° *p* < 0.001 versus vehicle; # *p* < 0.05 versus PEA; ### *p* < 0.001 versus PEA.

**Figure 5:**
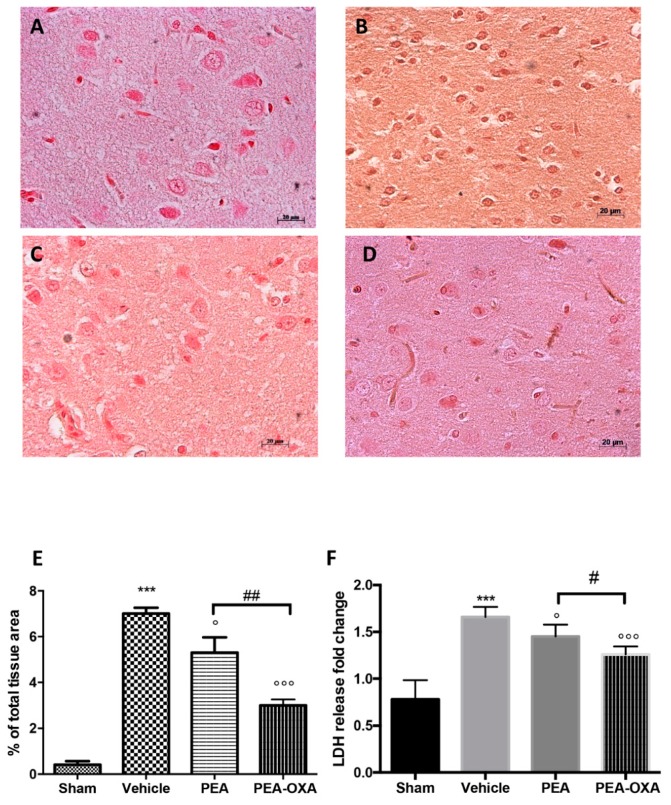
Efficacy of PEA-OXA on caspase 3 expression and LDH levels (induced by transient MCAo). An immunohistochemical analysis of caspase 3 (**E**) was performed. A marked increase in its expression was detected in brain samples from animals administered vehicle (**B**) compared to the sham-treated animals (**A**). This expression was notably reduced by treatment with PEA-OXA (**D**), while PEA treatment displayed less efficacy (**C**). PEA-OXA administration also reduced LDH levels to normal levels (**F**). For the immunohistochemistry, a 20× magnification is shown (50-µm scale bar). A *p*-value of less than 0.05 was considered significant: ° *p* < 0.05 versus vehicle; *** *p* < 0.001 versus sham; °°° *p* < 0.001 versus vehicle; # *p* < 0.05 versus PEA; ## *p* < 0.01 versus PEA.

The apoptotic pathway was evaluated by western blot analysis of Bax and Bcl-2 and by TUNEL staining: 24 h after transient MCAo, an upregulated expression of Bax (Figure 4A and the densitometric analysis in Figure 4B) and a reduced expression of Bcl-2 (Figure 4C and the densitometric analysis in Figure 4D) were detected in samples from vehicle-treated animals compared to the control group. PEA-OXA administration both significantly decreased Bax and increased Bcl-2 levels; in contrast, treatment with PEA at the same dose was less beneficial (Figure 4A,C and the densitometric analysis in Figure 4B,D). The same result was observed in TUNEL staining, where the number of positive cells was significantly diminished after treatment with PEA-OXA compared to the MCAo group (Figure 4E–I). Moreover, since it is well known that caspases play pivotal roles in apoptosis, we also observed the expression of caspases by immunostaining. The results obtained showed significantly increased levels of caspase 3 in the MCAo group compared to the sham group (Figure 5A and the densitometric analysis in Figure 5E). Treatment with PEA-OXA markedly reduced its expression (Figure 5D and the densitometric analysis in Figure 5E). PEA treatment showed less efficacy (Figure 5C and the densitometric analysis in Figure 5E). We also investigated the levels of LDH in serum. It was higher in the vehicle-treated animals compared to the sham, while PEA-OXA administration reduced its value (Figure 5F). Treatment with PEA at the same dose was less beneficial (Figure 5F).

### 2.5. Effect of PEA-OXA Treatment on MCAo-Induced IκB-α Degradation, NF-κB Translocation, and TGF-β Expression

**Figure 6:**
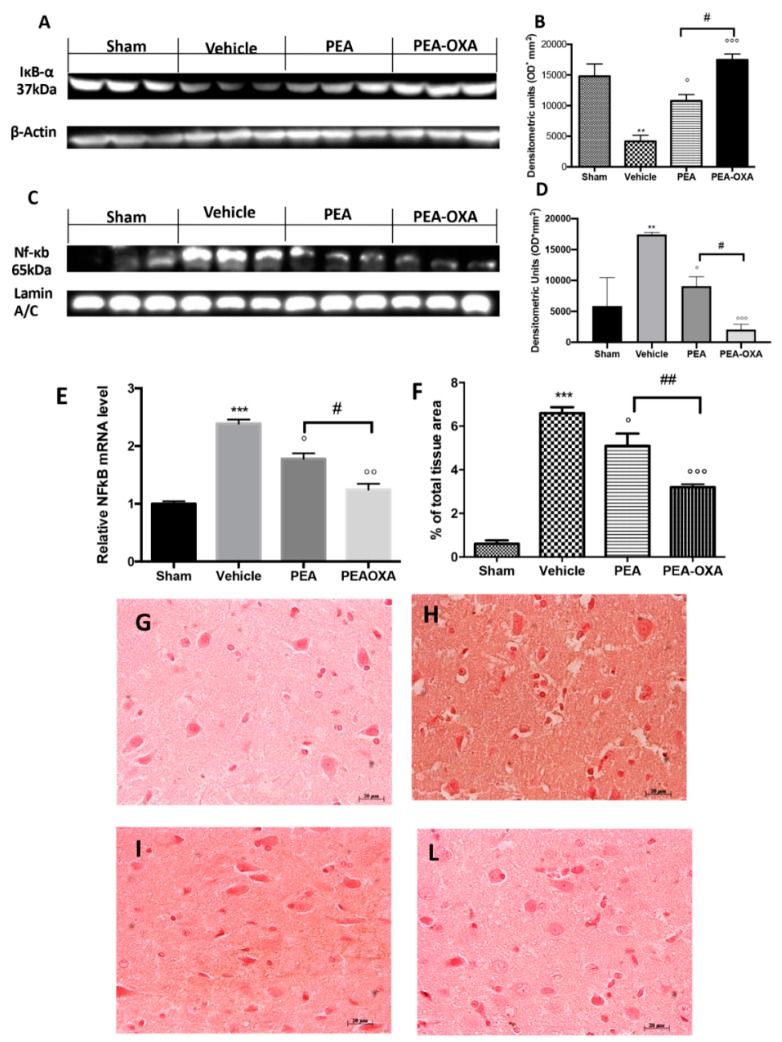
Efficacy of PEA-OXA on IκB-α degradation, NF-κB translocation, and TGF-β expression (induced by transient MCAo). Samples from vehicle-treated rats subjected to ischemia/reperfusion injury showed increased IκB-α degradation (**A**,**B**) and NF-κB translocation into the nucleus (**C**,**D**) compared to the sham-operated animals. PEA-OXA administration was able to significantly restore them to basal levels, while PEA showed less efficacy. Real-time PCR analysis of the NF-κB gene displayed the same trend (**E**). An immunohistochemical analysis TGF-β (**F**) was performed. A marked increase in its expression was detected in brain samples from animals administered vehicle (**F**,**H**) compared to the sham-treated animals (**F**,**G**). This expression was notably reduced by treatment with PEA-OXA (**F**,**L**). Treatment with PEA at the same dose was less beneficial (**F**,**I**). For the immunohistochemistry, a 20× magnification is shown (50-µm scale bar). A *p*-value of less than 0.05 was considered significant: ° *p* < 0.05 versus vehicle; ** *p* < 0.01 versus sham; °° *p* < 0.01 versus vehicle; *** *p* < 0.001 versus sham; °°° *p* < 0.001 versus vehicle; # *p* < 0.05 versus PEA; ## *p* < 0.01 versus PEA.

We found that IκB-α was significantly downregulated in vehicle-treated animals subjected to MCAo compared to the sham group. PEA-OXA administration partially restored this expression (Figure 6A and the densitometric analysis in Figure 6B), while PEA treatment displayed less efficacy (Figure 6A and the densitometric analysis in Figure 6B). Treatment with PEA-OXA also diminished NF-κB translocation into the nucleus compared to the vehicle-treated rats (Figure 6C and the densitometric analysis in Figure 6D), while PEA administration reduced its activation with significantly less efficacy (Figure 6C and the densitometric analysis in Figure 6D). NF-κB gene expression was increased in the vehicle-administered animals compared to the sham group, while PEA-OXA-treated animals showed reduced expression. In addition, PEA administration reduced NF-κB gene expression but with less efficacy (Figure 6E). Considering that recent evidence has demonstrated that TGF-β plays a key role in the formation of cerebral edema as well as in the neuronal processes that lead to necrosis in the acute stage of ischemia, we investigated the effect of PEA-OXA treatment on TGF-β expression after MCAo [47,48]. An immunohistochemical analysis of TGF-β expression showed increased staining in tissue sections from vehicle-treated animals (Figure 6H and the densitometric analysis in Figure 6F), which was reduced in samples from PEA-OXA-administered animals (Figure 6L and the densitometric analysis in Figure 6F). PEA administration was also able to reduce TGF-β expression (Figure 6I and the densitometric analysis in Figure 6F), but PEA-OXA treatment was more effective. The basal staining of TGF-β expression (Figure 6G and the densitometric analysis in Figure 6F) was detected in the sham-operated animals.

### 2.6. Effect of PEA-OXA Treatment on MCAo-Induced Cytokine Production 

**Figure 7:**
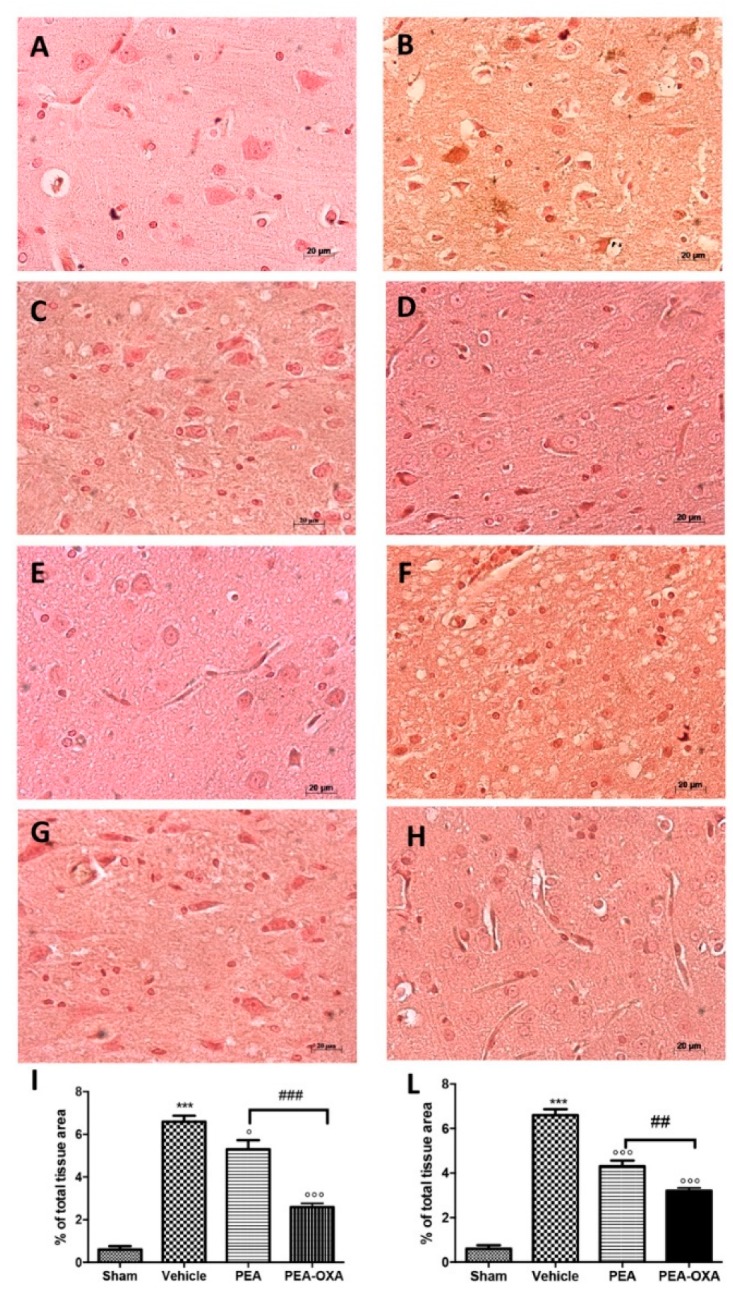
Efficacy of PEA-OXA on TNF-α and IL-1β expression (induced by transient MCAo). Twenty-four hours after transient MCAo, an increased expression of TNF-α (**B**,**I**) and IL-1β (**F**,**L**) was found in sections obtained from vehicle-treated rats compared to the sham-operated animals (**A**,**I**; **E**,**L**). PEA-OXA administration reduced both TNF-α (**D**,**I**) and IL-1β (**H**,**L**) expression more effectively than PEA did (**C**,**D**; **G**,**L**). For the immunohistochemistry, a 20× magnification is shown (50-µm scale bar). A *p*-value of less than 0.05 was considered significant: ° *p* < 0.05 versus vehicle; *** *p* < 0.001 versus sham; °°° *p* < 0.001 versus vehicle; ## *p* < 0.01 versus PEA; ### *p* < 0.001 versus PEA.

It is well known that TNF-α and IL-1 β have been implicated in the functional consequences of neuroinflammation. An immunohistochemical analysis of TNF-α and IL-1 β expression showed increased staining in tissue sections from vehicle-treated animals (Figure 7B,F, see densitometric analysis in Figure 7I,L), which was reduced in samples from PEA-OXA-administered animals (Figure 7D,H, see densitometric analysis in Figure 7I,L). PEA administration was also able to reduce TNF-α and IL-1β expression (Figure 7C,G, see densitometric analysis in Figure 7I,L), but PEA-OXA treatment was more effective. The basal staining of TNF-α and IL-1β (Figure 7A,E, see densitometric analysis in Figure 7I,L) was detected in sham-operated animals.

### 2.7. Effect of PEA-OXA Treatment on MCAo-Induced Reduced BDNF and GDNF Expression

**Figure 8:**
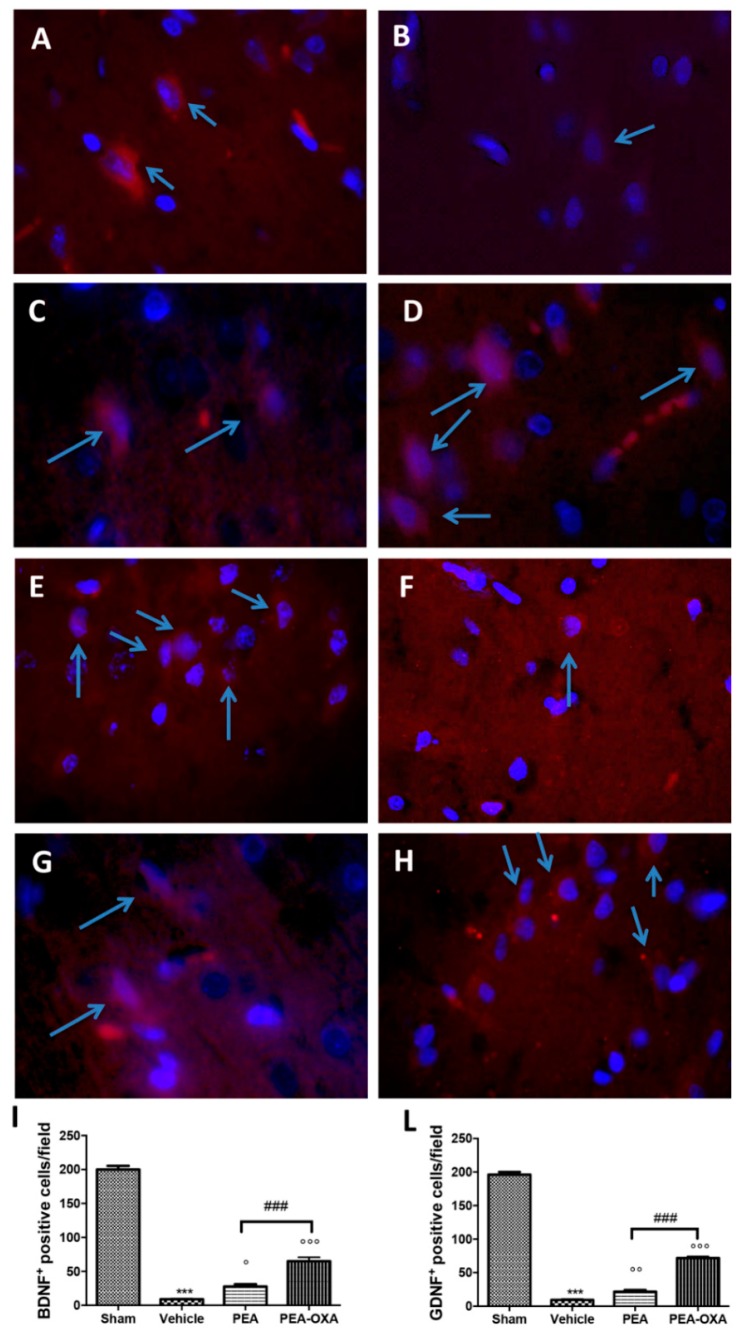
Efficacy of PEA-OXA on BDNF and GDNF expression (induced by transient MCAo). The immunofluorescence of brain samples showed that PEA-OXA-administered animals exhibited the presence of an increased number of positive cells for BDNF (**D**,**I**) and GDNF (**H**,**L**) staining compared to the samples from vehicle-treated rats (**B**,**I**; **F**,**L**). PEA displayed less protection (**C**,**I**; **G**,**L**). Sham-operated animals showed basal levels of BDNF (**A**,**I**) and GDNF (**E**,**L**). The number of BDNF- and GDNF-positive cells was counted in three sections per animal and is presented as the number of positive cells per high-power field. For immunofluorescence, a 100× magnification is shown (10-µm scale bar). A *p*-value of less than 0.05 was considered significant: ° *p* < 0.05 versus vehicle; °° *p* < 0.01 versus vehicle; *** *p* < 0.001 versus sham; °°° *p* < 0.001 versus vehicle; ### *p* < 0.001 versus PEA

Transient MCAo induced a marked reduction in BDNF and GDNF neurotrophic factors (Figure 8B,F, see densitometric analysis in Figure 8I,L). On the other hand, an immunofluorescence analysis of brain samples from PEA-OXA-treated animals showed a significantly increased expression of these factors (Figure 8D,H, see densitometric analysis in Figure 8I,L), while PEA displayed less protection (Figure 8C,G, see densitometric analysis in Figure 8I,L). The basal expression of BDNF and GDNF was found in tissues from control animals (Figure 8A,E, see densitometric analysis in Figure 8I,L).

## 3. Discussion

Cerebral ischemia is associated with a severe morbidity and high mortality rate, in particular in diabetic patients. Patients display related neurological disorders, such as cognitive and severe motor impairments [49,50]. Investigation of the mechanisms involved in worsening neuroinflammatory injury following cerebral ischemia in diabetes and linked hypoglycemia is very important. Suppressing potential candidates involved in enriching neuroinflammatory reactions may help to downgrade stroke severity and promote recovery in diabetic/hypoglycemic conditions [51]. It has been described that cerebral ischemia affects adaptive and innate immune systems [52]; leads to oxidative damage [53], excitotoxicity [54], blood–brain barrier dysfunction [55], microvascular injury [56], and post-ischemic inflammation [8,57,58,59]; and induces neuronal apoptosis [60]. Further investigations to discover new therapeutic strategies remain a high priority. Palmitoylethanolamide (PEA) is an important endogenous molecule that controls tissue reactivity and associated inflammatory antalgic phenomena, both in the central nervous system (CNS) and in innervated peripheral tissues [61,62]. *N*-acylethanolamines (NAEs), the family of molecules to which PEA belongs, are selectively degraded by two intracellular enzymes: *N*-acylethanolamine-hydrolyzing acid amidase (NAAA) [63,64] and fatty-acid amide hydrolase (FAAH) [65]. In particular, the amidase NAAA is the most important for PEA degradation [66], which means that the inhibition of this enzyme should increase PEA tissue levels [38,41,67,68]. Interesting strategies to modulate substrate availability underline the ability of the oxazoline derivatives of fatty acids to inhibit NAAA and display inhibitory activity toward inflammatory processes [69]. Recent evidence has shown the capability of a new PEA derivate, PEA-oxazoline (PEA-OXA), in inhibiting NAAA and decreasing inflammation processes [43,70,71,72]. In the present study, we focused our attention on the neuroprotective effects of PEA-OXA in a rat model of transient middle cerebral artery occlusion (MCAo) in diabetic rats. Our findings showed that oral administration of PEA-OXA at a dose of 10 mg/kg could significantly reduce lesion size, histological damage, and mast cell activation and degranulation associated with ischemia/reperfusion (I/R) injury. The inflammatory response to brain injury is characterized by a synchronized activation of various pathways that manage the expression of both anti- and proinflammatory mediators recruited from the blood and resident in tissue cells. In vitro studies have underlined a neuroprotective role for silent information regulator 1 (SIRT1) in ischemic injury [73]. Moreover, it is well known that PEA and SIRT1 share the same mechanism, expounding anti-inflammatory, analgesic, and neuroprotective effects [74,75,76,77]. In the present study, for the first time we show that PEA-OXA also has neuroprotective effects through the activation of SIRT1. During ischemia/reperfusion injury, the upregulation of SIRT1 is accompanied by the upregulation of mitochondrial uncoupling protein-2 (UCP2) [78,79]. PEA-OXA administration activated SIRT1 signaling and caused a reduced expression of UCP2. It is a proton channel of the inner mitochondrial membrane that regulates oxidative stress and energy supply [80] by modulating the oxidation of NADH, the level of ATP, and the redox balance [81,82]. An ischemic condition influenced the redox status, increasing the NAD^+^/NADH ratio and reducing SOD activity and the GSH level. SIRT1 activation induced by PEA-OXA administration caused a decrease in the NAD^+^/NADH ratio and an increase in SOD activity and the GSH level. It is well described that brain ischemia/reperfusion injury leads to the activation of the apoptotic pathway [83]. The activation of SIRT1 signaling through PEA-OXA administration increased the expression of B-cell lymphoma 2 (Bcl-2) and decreased the expression of Bax. It also decreased the expression of caspase 3, TUNEL staining, and LDH in serum. PEA-OXA, targeting SIRT1, mediated different effects that contributed to a protective effect of the nervous system. SIRT1 has been described as interacting with NF-κB and preventing its transcriptional activity [84]. It is one of the main pathways activated by ischemic brain injury [14,15]. Normally, it is sequestered into the cytoplasm by the inhibitor protein IkBα. Once IkBα is degraded, it allows for NF-κB translocation into the nucleus [85]. NF-κB, in turn, induces the expression of proinflammatory genes, including chemokines, cytokines, and adhesion molecules [86]. PEA-OXA administration reduced IκB-α degradation and consequently NF-κB translocation into the nucleus. Moreover, our results clearly demonstrate that PEA-OXA treatment downregulated the IL-1β, TGF-β, and TNF-α expression induced by cerebral ischemia [48]. In addition, neurotrophic family proteins exerted neuroprotective effects against ischemic stress, protecting against brain damage induced by ischemia and alleviating cognitive impairment [87]. Following MCAo, BDNF and GDNF expression was downregulated [88], while PEA-OXA administration could significantly restore their expression to sham levels.

## 4. Materials and Methods

### 4.1. Animals

Male Wistar rats (Envigo, Milan, Italy) weighing 200–250 g were employed. Animals were divided in groups of three with free access to water and food under standardized humidity and temperature. This study was approved by the University of Messina Review Board for the care of animals (Protocol number 8/U-apr16). Animal care was in conformity with current legislation for the protection of animals used for scientific purposes (Directive 2010/63/EU, 9 April 2016) and the ARRIVE guidelines.

### 4.2. Middle Cerebral Artery Occlusion

Focal cerebral ischemia was induced by transient MCAo in the right hemisphere. The animals were anesthetized with inhaled 1.0–2.0% isoflurane and 5.0% isoflurane (Baxter International) in air through the use of a mask. Body temperature was preserved at 37 °C with a heating pad and was supervised via an intrarectal type T thermocouple (Harvard, Kent, UK). The animals were located in a stereotaxic system (Kopf). Middle cerebral artery (MCA) occlusion was provided by introducing a 4–0 nylon monofilament (Ethilon; Johnson & Johnson, Somerville, NJ, USA), precoated with silicone (Xantopren; Heraeus Kulzer, Germany) via the external carotid artery into the internal carotid artery to occlude the MCA [89,90,91]. Sham animals were subjected to the same procedure, but the filament was introduced into the internal carotid artery and suddenly withdrawn. At the end of the procedure, anesthesia was discontinued, and the rats were returned to a prone position. Laser Doppler flowmetry (PeriFlux System 5000; Perimed AB, Stockholm, Sweden) with a flexible probe over the skull was used to monitor regional cerebral blood flow (rCBF), as previously described [92].

### 4.3. Synthesis of PEA and PEA-OXA

PEA and PEA-OXA were synthesized as previously described by Impellizzeri and colleagues [43].

### 4.4. Induction of Diabetes

Diabetes was induced by streptozotocin, as previously described by Di Paola et al. [93]. We confirmed a diabetic condition by evaluating glucose levels at 15 and 60 days through a blood glucose meter (Table 1) (Accu-Check Active^®^; Roche Diagnostic, Milan, Italy).

### 4.5. Experimental Groups

Sixty days after the induction of diabetes, rats were randomly assigned into different groups, as described below (*n* = 30):

TZ-hyperglycemic rats (ischemia/reperfusion (I/R) + vehicle): Diabetic rats were subjected to MCAo (1 h) followed by 24 h of reperfusion [94]. One hour after ischemia and six hours after reperfusion, carboxymethyl cellulose (CMC) in saline (1.5 %, *w*/*v*) was administered (os), and the rats were sacrificed 24 h later.

STZ-hyperglycemic rats (I/R + PEA): Diabetic rats were subjected to the surgical procedures described above. PEA (10 mg/kg in 1.5% CMC) was administered (os) 1 h after ischemia and 6 h after reperfusion, and the rats were sacrificed 24 h later;

STZ-hyperglycemic rats (I/R + PEA-OXA): Diabetic rats were subjected to the surgical procedures described above. PEA-OXA (10 mg/kg in 1.5% CMC) was administered (os) 1 h after ischemia and 6 h after reperfusion, and the rats were sacrificed 24 h later;

Sham + vehicle: Rats were subjected to the same procedures, but the filament was introduced into the internal carotid artery and suddenly withdrawn, and the rats were kept under anesthesia for the duration of the experiment. The animals were administered (os) 1.5% (*w*/*v*) CMC in saline at the same time point as the MCAo group and were sacrificed 24 h later;

Sham + PEA: The animals were the same as the sham-operated rats except for the administration of PEA (10 mg/kg in 1.5% CMC, os) 1 h after ischemia and 6 h after reperfusion, and the rats were sacrificed 24 h later (data not shown);

Sham + PEA-OXA: The animals were the same as the sham-operated rats except for the administration of PEA-OXA (10 mg/kg in 1.5% CMC, os) 1 h after ischemia and 6 h after reperfusion, and they were sacrificed 24 h later (data not shown).

Rats were given buprenorphine (0.03 mg/kg, subcutaneous) and saline immediately after surgery.

Randomization based on a single sequence of random assignments is known as simple randomization [95,96,97]. This technique maintains complete randomness of the assignment of a subject to a particular group. We used the most common and basic method of simple randomization: flipping a coin.

The doses (10 mg/kg) of PEA and PEA-OXA, the administration route (os), and the vehicle employed were chosen basing on our previous study [44,72,89].

At the conclusion of the experiment, animals were euthanized under anesthesia: the brain was removed and fixed in 10% neutral-buffered formalin and then embedded in paraffin for future histological analysis or stored at −80 °C for western blot or biochemical analyses (see graphical timeline).

### 4.6. Quantification of Infarct Volume

Tissues were incubated in a 2% solution of 2,3,5-triphenyltetrazolium chloride (TTC) for 30 min at 37 °C, processed, and quantified as previously described [96,97,98]. All analyses were carried out by two observers blinded to the treatment.

### 4.7. Histological Evaluation

A histological evaluation was made as previously described by Ahmad et al. [88,99]. For the histology, a 20× magnification is shown (50-µm scale bar). All analyses were carried out by two observers blinded to the treatment.

### 4.8. Toluidine Blue Staining

In order to evaluate mast cell numbers and degranulation, brain tissue sections were stained with toluidine blue. Sections were stained blue, while the mast cells were stained purple. The mast cell count was performed on each slide through an Axiovision Zeiss (Milan, Italy) microscope and is shown at a 100× magnification (10-µm scale bar). All analyses were carried out by two observers blinded to the treatment.

### 4.9. Immunohistochemical Localization of Tryptase, Caspase-3, Interleukin-1 Beta (IL-1β), Tumor Necrosis Factor-Alpha (TNF-α), and Transforming Growth Factor Beta (TGF-β)

An immunohistochemical localization was done as previously described by Cordaro et al. [45]. Slices were incubated at room temperature overnight with one of the following primary antibodies: anti-tryptase (Santa Cruz Biotechnology, Heidelberg, Germany, 1:100 in PBS, *v*/*v*), anti-caspase-3 (Cell Signalling, Danvers, MA, USA 1:300 in PBS, *v*/*v*), anti-IL-1β (Santa Cruz Biotechnology, Heidelberg, Germany, 1:50 in PBS, *v*/*v*), anti-TNF-α (Santa Cruz Biotechnology, Heidelberg, Germany, 1:100 in PBS, *v*/*v*), or anti-TGF-β (Santa Cruz Biotechnology, Heidelberg, Germany 1:50 in PBS, *v*/*v*). For a graphic display of the densitometric analyses, the % of positive staining (brown staining) was measured by computer-assisted color image analysis (Leica QWin V3, Newcastle, UK). The percentage area of immunoreactivity (determined by the number of positive pixels) was expressed as % of total tissue area (red staining) within five random fields at a 40× magnification. In particular, first the colors of the images that were stained on the molecule of interest were defined. Once these colors were defined, they were automatically detected in all samples. This is a semiquantitative analysis that measures areas and not intensities [100,101]. Replicates for each experimental condition and histochemical staining were acquired from each mouse in each experimental group. All immunohistochemical analyses were carried out by two observers blinded to the treatment.

### 4.10. Immunofluorescence of Brain-Derived Neurotrophic Factor (BDNF) and Glial Cell-derived Neurotrophic Factor (GDNF)

Brain tissue sections were incubated with one of the following primary antibodies—anti-BDNF rabbit polyclonal (1:100, Santa Cruz Biotechnology) or anti-GDNF (1:100, Santa Cruz Biotechnology)—in a humidified chamber at 37 °C overnight. Sections were washed with PBS and were incubated with secondary antibody TEXAS RED-conjugated antirabbit Alexa Fluor-594 antibody (1:1000 in PBS, *v*/*v*, Molecular Probes, Altrincham, UK) and with FITC-conjugated antimouse Alexa Fluor-488 antibody (1:2000 *v*/*v*, Molecular Probes, Altrincham, UK) for 1 h at 37 °C. Sections were laved, and for nuclear staining, 4′,6′-diamidino-2-phenylindole (DAPI; Hoechst, Frankfurt, Germany) (2 μg/mL) in PBS was added. Sections were observed and photographed at a 100× magnification using a Leica DM2000 microscope. All analyses were carried out by two observers blinded to the treatment. For immunofluorescence, a 100× magnification is shown (10-µm scale bar).

### 4.11. Western Blot Analysis for SIRT1, UCP2, IkB-α, NF-kB, Bax, and Bcl-2

Western blots were done as previously described [45]. Filters were blocked with 1 × PBS and 5% (*w*/*v*) no-fat dried milk (PM) for 40 min at room temperature and then probed with one of the following primary antibodies—anti-SIRT1 (1:500, Santa Cruz Biotechnology), anti-UCP2 (1:500, Santa Cruz Biotechnology), anti-IkB-α (1:500, Santa Cruz Biotechnology, #sc1643), anti-Bax (1:500, Santa Cruz Biotechnology, #sc526), or anti-Bcl-2 (1:500, Santa Cruz Biotechnology, #sc492)—in 1× PBS, 0.1% Tween-20, and 5% *w*/*v* no-fat dried milk (PMT) at 4 °C overnight. Membranes were incubated with peroxidase-conjugated bovine antimouse IgG secondary antibody or peroxidase-conjugated goat antirabbit IgG (1:2000, Jackson ImmunoResearch, West Grove, PA,USA) for 1 h at room temperature. Blots were also incubated with primary antibody against β-actin protein (1:10,000; Sigma-Aldrich Corp., St. Louis, MO, USA) or laminin (1:10,000; Sigma-Aldrich Corp, St. Louis, MO, USA), which were used as internal standards. The relative expressions of the protein bands of SIRT1 (120 kDa), UCP2 (33 kDa), IkB-α (37 kDa), NF-kB p65 (65 kDa), Bax (23 kDa), and Bcl-2 (29 kDa) were detected and quantified by densitometry. In the experiments, including the western blot analysis, a representative blot is displayed and a densitometric analysis is related in each figure. All analyses were carried out by two observers blinded to the treatment.

### 4.12. TUNEL Staining

The TUNEL staining protocol was according to the Roche protocol previously described by Fusco et al. [102]. The tissue was then rinsed in PBS 3 times for 5 min and then observed using an excitation wavelength in the range of 520–560 nm (maximum 540; green) and in the range of 570–620 nm (maximum 580 nm; red). For TUNEL staining, a 100× magnification is shown (10-µm scale bar). All analyses were carried out by two observers blinded to the treatment.

### 4.13. NAD+/NADH Assay

NAD+ and NADH levels were analyzed by an assay kit according to the manufacturer’s instructions (Suzhou Comin, China). The brain was homogenized in the lysis buffer and was then centrifuged at 4 °C. MTT was then reduced to formazan by the supernatant. The formazan was dissolved in the buffer, and the absorbance was read at 570 nm. The relative level of NAD+ was expressed as an NAD+/NADH ratio. All analyses were carried out by two observers blinded to the treatment.

### 4.14. GSH Levels Assay

GSH levels were analyzed using an assay kit according to the manufacturer’s instructions (Nanjing Jiancheng, Jiangsu, China). The brain was homogenized in saline and was then centrifuged at 4 °C. The supernatant was set to react with 5,5′-dithiobis(2-nitrobenzoic acid) (DTNB). Then the absorbance was read at 405 nm. The level of GSH was expressed as μmol/g protein. All analyses were carried out by two observers blinded to the treatment.

### 4.15. SOD Activity Assay

SOD activity was determined using an activity assay kit according to the manufacturer’s instructions. The brain was homogenized in saline and was then centrifuged at 4 °C. The absorbance was read at 450 nm. The SOD activity was expressed as U/mg protein. All analyses were carried out by two observers blinded to the treatment.

### 4.16. LDH Release Detection

Twenty-four hours after reperfusion, each rat was euthanized, the blood was collected, and the serum was separated. Lactate dehydrogenase (LDH) release was measured. The detection was conducted with an assay kit (Solarbio, Beijing, China). All analyses were carried out by two observers blinded to the treatment.

### 4.17. RT-PCR

Total RNA was extracted according to the manufacturer’s protocol and reverse-transcribed using 2-μg oligo(dT)15 primer, 10 units of AMV reverse transcriptase, 40 units of RNase inhibitor (all from Promega, Southampton, UK), and 1.25 mM of dNTP (Bioline, London, UK) for 45 min at 42 °C. Real-time PCR was carried out using TaqMan Universal PCR master mix and fluorescent primers obtained from Quiagen (QuantiTect primers, Skelton House, UK). Cycling conditions were set according to the manufacturer’s instructions. Sequence-specific fluorescent signals were detected by an ABI Prism 7700Sequence Detector System, and mRNA data were normalized relative to GADPH and then used to calculate expression levels. 

### 4.18. Materials

STZ and the other compounds were acquired from Sigma-Aldrich Company Ltd. (Milan, Italy). All other chemicals were of the highest viable grade available. All stock solutions were arranged in nonpyrogenic saline (0.9% NaCl; Baxter, Milan, Italy). PEA and PEA-OXA were kindly provided by the Epitech Group (Saccolongo, Padua, Italy).

### 4.19. Statistical Analysis

All values are shown as the mean ± standard error of the mean (SEM) of *N* observations. In the experiments, including the immunohistochemistry, histology, and immunofluorescence, the figures shown are representative of at least three experiments performed on different days on tissue sections collected from all animals in each group. Data were analyzed by one-way ANOVA followed by a Bonferroni post hoc test for multiple comparisons. A *p*-value of less than 0.05 was considered significant: * *p* < 0.05 versus sham; ° *p* < 0.05 versus vehicle; ** *p* < 0.01 versus sham; °° *p* < 0.01 versus vehicle; *** *p* < 0.001 versus sham; °°° *p* < 0.001 versus vehicle; # *p* < 0.05 versus PEA; ## *p* < 0.01 versus PEA; ### *p* < 0.001 versus PEA.

## 5. Conclusions

In summary, our data clearly demonstrated for the first time that PEA-OXA treatment (10 mg/kg) significantly improved neurological injuries induced by transient MCAo compared to a simple PEA treatment at the same dose.

## Figures and Tables

**Table 1:** Evaluation of glucose levels.

Experimental Group	Blood Glucose		
	Day 0	Day 15	Day 60
Sham	120 ± 3.1	136 ± 2.5	126 ± 3.0
I/R	130 ± 2.7	459 ± 2.9	455 ± 2.4
PEA	125 ± 2.5	463 ± 2.2	459 ± 2.5
PEA-OXA	131 ± 1.9	466 ± 2.7	462 ± 2.3

## Abbreviations

HE	hematoxylin and eosin
GFAP	antiglial fibrillary acidic protein
Iba-1	ionized calcium-binding adaptor molecule 1
BDNF	brain-derived neurotrophic factor
GDNF	glial cell-derived neurotrophic factor
IL-1β	interleukin 1 beta
IκB-α	nuclear factor of kappa light polypeptide gene enhancer in B-cells inhibitor alpha
NAAA	*N*-acylethanolamine-hydrolyzing acid amidase
NFκB	nuclear factor-κB
NGF	nerve growth factor
PEA	*N*-palmitoylethanolamide
PEA-OXA	2-pentadecyl-2-oxazoline
rCBF	regional cerebral blood flow
ROS	reactive oxygen species
SIRT1	silent information regulator 1
STZ	streptozotocin
TGF- β	transforming growth factor beta
TNF-α	tumor necrosis factor-alpha
UCP2	uncoupling protein 2
VEGF	vascular endothelial growth factor

## References

[1] Emerging Risk Factors C., Sarwar N., Gao P., Seshasai S.R., Gobin R., Kaptoge S., di Angelantonio E., Ingelsson E., Lawlor D.A., Selvin E. (2010). Diabetes mellitus, fasting blood glucose concentration, and risk of vascular disease: A collaborative meta-analysis of 102 prospective studies. Lancet.

[2] Bourne R.R., Stevens G.A., White R.A., Smith J.L., Flaxman S.R., Price H., Jonas J.B., Keeffe J., Leasher J., Naidoo K. (2013). Vision Loss Expert, G. Causes of vision loss worldwide, 1990-2010: A systematic analysis. Lancet Glob. Health.

[3] Biller J., Love B.B. (1993). Diabetes and stroke. Med. Clin. North. Am..

[4] Vinik A., Flemmer M. (2002). Diabetes and macrovascular disease. J. Diabetes Complicat..

[5] Iwata N., Takayama H., Xuan M., Kamiuchi S., Matsuzaki H., Okazaki M., Hibino Y. (2015). Effects of Etanercept against Transient Cerebral Ischemia in Diabetic Rats. Biomed. Res. Int..

[6] Jin Y., Silverman A.J., Vannucci S.J. (2009). Mast cells are early responders after hypoxia-ischemia in immature rat brain. Stroke; A J. Cereb. Circ..

[7] Silver R., Curley J.P. (2013). Mast cells on the mind: New insights and opportunities. Trends Neurosci..

[8] Wang Q., Tang X.N., Yenari M.A. (2007). The inflammatory response in stroke. J. Neuroimmunol..

[9] Kawabori M., Yenari M.A. (2015). Inflammatory responses in brain ischemia. Curr. Med. Chem..

[10] Bramlett H.M., Dietrich W.D. (2004). Pathophysiology of cerebral ischemia and brain trauma: Similarities and differences. J. Cereb. Blood Flow Metab..

[11] Salman M.M., Kitchen P., Woodroofe M.N., Bill R.M., Conner A.C., Heath P.R., Conner M.T. (2017). Transcriptome analysis of gene expression provides new insights into the effect of mild therapeutic hypothermia on primary human cortical astrocytes cultured under hypoxia. Front. Cell Neurosci..

[12] Ginsberg M.D. (2003). Adventures in the pathophysiology of brain ischemia: Penumbra, gene expression, neuroprotection: The 2002 Thomas Willis Lecture. Stroke.

[13] Thimmulappa R.K., Lee H., Rangasamy T., Reddy S.P., Yamamoto M., Kensler T.W., Biswal S. (2006). Nrf2 is a critical regulator of the innate immune response and survival during experimental sepsis. J. Clin. Investig..

[14] Barnes P.J., Karin M. (1997). Nuclear factor-kappaB: A pivotal transcription factor in chronic inflammatory diseases. New Engl. J. Med..

[15] Bowie A., O’Neill L.A. (2000). Oxidative stress and nuclear factor-kappaB activation: A reassessment of the evidence in the light of recent discoveries. Biochem. Pharmacol..

[16] Calabresi P., Cupini L.M., Centonze D., Pisani F., Bernardi G. (2003). Antiepileptic drugs as a possible neuroprotective strategy in brain ischemia. Ann. Neurol..

[17] ArunaDevi R., Lata S., Bhadoria B.K., Ramteke V.D., Kumar S., Sankar P., Kumar D., Tandan S.K. (2010). Neuroprotective effect of 5,7,3’,4’,5’-pentahydroxy dihydroflavanol-3-O-(2’’-O-galloyl)-beta-D-glucopyranoside, a polyphenolic compound in focal cerebral ischemia in rat. Eur. J. Pharm..

[18] ArunaDevi R., Ramteke V.D., Kumar S., Shukla M.K., Jaganathan S., Kumar D., Sharma A.K., Tandan S.K. (2010). Neuroprotective effect of s-methylisothiourea in transient focal cerebral ischemia in rat. Nitric Oxide Biol. Chem..

[19] Ye Q., Li Q., Zhou Y., Xu L., Mao W., Gao Y., Li C., Xu Y., Xu Y., Liao H. (2015). Synthesis and Evaluation of 3-(furo[2 –b]pyridin-3-yl)-4-(1H-indol-3-yl)-maleimides as Novel GSK-3beta Inhibitors and Anti-Ischemic Agents. Chem. Biol. Drug Des..

[20] Jin Z., Liang J., Wang J., Kolattukudy P.E. (2015). MCP-induced protein 1 mediates the minocycline-induced neuroprotection against cerebral ischemia/reperfusion injury in vitro and in vivo. J. Neuroinflammation.

[21] Corbett D., Jeffers M., Nguemeni C., Gomez-Smith M., Livingston-Thomas J. (2015). Lost in translation: Rethinking approaches to stroke recovery. Prog. Brain Res..

[22] Sughrue M.E., Grobelny B.T., Ducruet A.F., Komotar R.J., Mocco J., Sciacca R.R., Sander Connolly E. (2010). Data presentation in rodent stroke studies and the predictive value of confidence intervals. J. Clin. Neurosci.: Off. J. Neurosurg. Soc. Australas..

[23] Hussain M.S., Shuaib A. (2008). Research into neuroprotection must continue... But with a different approach. Stroke; A J. Cereb. Circ..

[24] Prestigiacomo C.J., Kim S.C., Connolly E.S., Liao H., Yan S.F., Pinsky D.J. (1999). CD18-mediated neutrophil recruitment contributes to the pathogenesis of reperfused but not nonreperfused stroke. Stroke A J. Cereb. Circ..

[25] Zhang L., Zhang Z.G., Zhang R.L., Lu M., Krams M., Chopp M. (2003). Effects of a selective CD11b/CD18 antagonist and recombinant human tissue plasminogen activator treatment alone and in combination in a rat embolic model of stroke. Stroke; A J. Cereb. Circ..

[26] Becker K.J. (2002). Anti-leukocyte antibodies: LeukArrest (Hu23F2G) and Enlimomab (R6.5) in acute stroke. Curr. Med. Res. Opin..

[27] Investigators E.A.S.T. (2001). Use of anti-ICAM-1 therapy in ischemic stroke results of the Enlimomab Acute Stroke Trial. Neurology.

[28] Pacher P., Batkai S., Kunos G. (2006). The endocannabinoid system as an emerging target of pharmacotherapy. Pharm. Rev..

[29] Lambert D.M., Vandevoorde S., Jonsson K.O., Fowler C.J. (2002). The palmitoylethanolamide family: A new class of anti-inflammatory agents?. Curr. Med. Chem..

[30] Buckley C.D., Gilroy D.W., Serhan C.N., Stockinger B., Tak P.P. (2013). The resolution of inflammation. Nat. Rev. Immunol..

[31] Skaper S.D., Facci L. (2012). Mast cell-glia axis in neuroinflammation and therapeutic potential of the anandamide congener palmitoylethanolamide. Philos. Trans. R. Soc. Lond. Ser. B Biol. Sci..

[32] Skaper S.D., Facci L., Giusti P. (2014). Mast cells, glia and neuroinflammation: Partners in crime?. Immunology.

[33] Impellizzeri D., Esposito E., Attley J., Cuzzocrea S. (2014). Targeting inflammation: New therapeutic approaches in chronic kidney disease (CKD). Pharmacol. Res..

[34] Alhouayek M., Muccioli G.G. (2014). Harnessing the anti-inflammatory potential of palmitoylethanolamide. Drug Discov. Today.

[35] Esposito E., Cordaro M., Cuzzocrea S. (2014). Roles of fatty acid ethanolamides (FAE) in traumatic and ischemic brain injury. Pharmacol. Res..

[36] Fidaleo M., Fanelli F., Ceru M.P., Moreno S. (2014). Neuroprotective properties of peroxisome proliferator-activated receptor alpha (PPARalpha) and its lipid ligands. Curr. Med. Chem..

[37] Ueda N., Tsuboi K., Uyama T. (2010). N-acylethanolamine metabolism with special reference to N-acylethanolamine-hydrolyzing acid amidase (NAAA). Prog. Lipid Res..

[38] Solorzano C., Zhu C., Battista N., Astarita G., Lodola A., Rivara S., Mor M., Russo R., Maccarrone M., Antonietti F. (2009). Selective N-acylethanolamine-hydrolyzing acid amidase inhibition reveals a key role for endogenous palmitoylethanolamide in inflammation. Proc. Natl. Acad. Sci. USA.

[39] Yamano Y., Tsuboi K., Hozaki Y., Takahashi K., Jin X.H., Ueda N., Wada A. (2012). Lipophilic amines as potent inhibitors of N-acylethanolamine-hydrolyzing acid amidase. Bioorganic Med. Chem..

[40] Yang L., Li L., Chen L., Li Y., Chen H., Li Y., Ji G., Lin D., Liu Z., Qiu Y. (2015). Potential analgesic effects of a novel N-acylethanolamine acid amidase inhibitor F96 through PPAR-alpha. Sci. Rep..

[41] Ribeiro A., Pontis S., Mengatto L., Armirotti A., Chiurchiu V., Capurro V., Fiasella A., Nuzzi A., Romeo E., Moreno-Sanz G. (2015). A Potent Systemically Active N-Acylethanolamine Acid Amidase Inhibitor that Suppresses Inflammation and Human Macrophage Activation. Acs Chem. Biol..

[42] Skaper S.D., Facci L., Barbierato M., Zusso M., Bruschetta G., Impellizzeri D., Cuzzocrea S., Giusti P. (2015). N-Palmitoylethanolamine and Neuroinflammation: A Novel Therapeutic Strategy of Resolution. Mol. Neurobiol..

[43] Impellizzeri D., Cordaro M., Bruschetta G., Crupi R., Pascali J., Alfonsi D., Marcolongo G., Cuzzocrea S. (2016). 2-pentadecyl-2-oxazoline: Identification in coffee, synthesis and activity in a rat model of carrageenan-induced hindpaw inflammation. Pharm. Res..

[44] Impellizzeri D., Cordaro M., Bruschetta G., Siracusa R., Crupi R., Esposito E., Cuzzocrea S. (2017). N-Palmitoylethanolamine-Oxazoline as a New Therapeutic Strategy to Control Neuroinflammation: Neuroprotective Effects in Experimental Models of Spinal Cord and Brain Injury. J. Neurotrauma.

[45] Cordaro M., Siracusa R., Crupi R., Impellizzeri D., Peritore A.F., D’Amico R., Gugliandolo E., di Paola R., Cuzzocrea S. (2018). 2-Pentadecyl-2-Oxazoline Reduces Neuroinflammatory Environment in the MPTP Model of Parkinson Disease. Mol. Neurobiol..

[46] Dong H., Wang Y., Zhang X., Zhang X., Qian Y., Ding H., Zhang S. (2019). Stabilization of Brain Mast Cells Alleviates LPS-Induced Neuroinflammation by Inhibiting Microglia Activation. Front. Cell Neurosci.

[47] Yao X., Miao W., Li M., Wang M., Ma J., Wang Y., Miao L., Feng H. (2010). Protective effect of albumin on VEGF and brain edema in acute ischemia in rats. Neurosci Lett.

[48] Ali C., Docagne F., Nicole O., Lesne S., Toutain J., Young A., Chazalviel L., Divoux D., Caly M., Cabal P. (2001). Increased expression of transforming growth factor-beta after cerebral ischemia in the baboon: An endogenous marker of neuronal stress?. J. Cereb Blood Flow Metab.

[49] Chu H.X., Kim H.A., Lee S., Broughton B.R., Drummond G.R., Sobey C.G. (2016). Evidence of CCR2-independent transmigration of Ly6C(hi) monocytes into the brain after permanent cerebral ischemia in mice. Brain Res..

[50] Shi S.S., Yang W.Z., Chen Y., Chen J.P., Tu X.K. (2014). Propofol reduces inflammatory reaction and ischemic brain damage in cerebral ischemia in rats. Neurochem. Res..

[51] Shukla V., Shakya A.K., Perez-Pinzon M.A., Dave K.R. (2017). Cerebral ischemic damage in diabetes: An inflammatory perspective. J. Neuroinflammation.

[52] Famakin B.M. (2014). The immune response to acute focal cerebral ischemia and associated post-stroke immunodepression: A focused review. Aging Dis..

[53] Chen H., Yoshioka H., Kim G.S., Jung J.E., Okami N., Sakata H., Maier C.M., Narasimhan P., Goeders C.E., Chan P.H. (2011). Oxidative stress in ischemic brain damage: Mechanisms of cell death and potential molecular targets for neuroprotection. Antioxid. Redox Signal..

[54] Paschen W. (1996). Glutamate excitotoxicity in transient global cerebral ischemia. Acta. Neurobiol. Exp..

[55] Abdullahi W., Tripathi D., Ronaldson P.T. (2018). Blood-brain barrier dysfunction in ischemic stroke: Targeting tight junctions and transporters for vascular protection. Am. J. Physiol. -Cell Physiol..

[56] Logsdon A.F., Lucke-Wold B.P., Turner R.C., Huber J.D., Rosen C.L., Simpkins J.W. (2011). Role of microvascular disruption in brain damage from traumatic brain injury. Compr. Physiol..

[57] Lai T.W., Zhang S., Wang Y.T. (2014). Excitotoxicity and stroke: Identifying novel targets for neuroprotection. Prog. Neurobiol..

[58] Trendelenburg G. (2014). Molecular regulation of cell fate in cerebral ischemia: Role of the inflammasome and connected pathways. J. Cereb. Blood Flow Metab: Off. J. Int. Soc. Cereb. Blood Flow Metab..

[59] Moretti R., Pansiot J., Bettati D., Strazielle N., Ghersi-Egea J.F., Damante G., Fleiss B., Titomanlio L., Gressens P. (2015). Blood-brain barrier dysfunction in disorders of the developing brain. Front. Neurosci..

[60] Iadecola C., Anrather J. (2011). The immunology of stroke: From mechanisms to translation. Nat. Med..

[61] Costa B., Conti S., Giagnoni G., Colleoni M. (2002). Therapeutic effect of the endogenous fatty acid amide, palmitoylethanolamide, in rat acute inflammation: Inhibition of nitric oxide and cyclo-oxygenase systems. Br. J. Pharmacol..

[62] Lo Verme J., Fu J., Astarita G., La Rana G., Russo R., Calignano A., Piomelli D. (2005). The nuclear receptor peroxisome proliferator-activated receptor-alpha mediates the anti-inflammatory actions of palmitoylethanolamide. Mol. Pharmacol..

[63] Ueda N., Yamanaka K., Yamamoto S. (2001). Purification and characterization of an acid amidase selective for N-palmitoylethanolamine, a putative endogenous anti-inflammatory substance. J. Biol. Chem..

[64] Tsuboi K., Takezaki N., Ueda N. (2007). The N-acylethanolamine-hydrolyzing acid amidase (NAAA). Chem. Biodivers..

[65] McKinney M.K., Cravatt B.F. (2005). Structure and function of fatty acid amide hydrolase. Annu. Rev. Biochem..

[66] Tai T., Tsuboi K., Uyama T., Masuda K., Cravatt B.F., Houchi H., Ueda N. (2012). Endogenous molecules stimulating N-acylethanolamine-hydrolyzing acid amidase (NAAA). Acs Chem. Neurosci..

[67] Alhouayek M., Bottemanne P., Subramanian K.V., Lambert D.M., Makriyannis A., Cani P.D., Muccioli G.G. (2015). N-Acylethanolamine-hydrolyzing acid amidase inhibition increases colon N-palmitoylethanolamine levels and counteracts murine colitis. Faseb J. Off. Publ. Fed. Am. Soc. Exp. Biol..

[68] Petrosino S., Ahmad A., Marcolongo G., Esposito E., Allarà M., Verde R., Cuzzocrea S., Di Marzo V. (2015). Diacerein is a potent and selective inhibitor of palmitoylethanolamide inactivation with analgesic activity in a rat model of acute inflammatory pain. Pharmacol. Res..

[69] Della Valle F., Della Valle M.F., Marcolongo G., Di Marzo V., Cuzzocrea S. (2016). Compositions and Methods for the Modulation of Specific Amidases for n-Acylethanolamines for Use in the Therapy of Inflammatory Diseases. Google Patents.

[70] Bandiera T., Ponzano S., Piomelli D. (2014). Advances in the discovery of N-acylethanolamine acid amidase inhibitors. Pharmacol. Res..

[71] Migliore M.D., Pontis S.D., Fuentes de Arriba A.L., Realini N., Torrente E., Armirotti A., Romeo E., Di Martino S., Russo D., Pizzirani D. (2016). Second-Generation Non-Covalent NAAA Inhibitors are Protective in a Model of Multiple Sclerosis. Angew. Chem..

[72] Xu J., Jackson C.W., Khoury N., Escobar I., Perez-Pinzon M.A. (2018). Brain SIRT1 Mediates Metabolic Homeostasis and Neuroprotection. Front. Endocrinol.

[73] Smart D., Jonsson K.O., Vandevoorde S., Lambert D.M., Fowler C.J. (2002). ‘Entourage’ effects of N-acyl ethanolamines at human vanilloid receptors. Comparison of effects upon anandamide-induced vanilloid receptor activation and upon anandamide metabolism. Brit. J. Pharm..

[74] Mazzari S., Canella R., Petrelli L., Marcolongo G., Leon A. (1996). N-(2-Hydroxyethyl)hexadecanamide is orally active in reducing edema formation and inflammatory hyperalgesia by down-modulating mast cell activation. Eur J. Pharm..

[75] Farquhar-Smith W.P., Rice A.S.C. (2001). Administration of endocannabinoids prevents a referred hyperalgesia associated with inflammation of the urinary bladder. Anesthesiology.

[76] Picard F., Kurtev M., Chung N.J., Topark-Ngarm A., Senawong T., de Oliveira R.M., Leid M., McBurney M.W., Guarente L. (2004). Sirt1 promotes fat mobilization in white adipocytes by repressing PPAR-gamma. Nature.

[77] De Bilbao F., Arsenijevic D., Vallet P., Hjelle O.P., Ottersen O.P., Bouras C., Raffin Y., Abou K., Langhans W., Collins S. (2004). Resistance to cerebral ischemic injury in UCP2 knockout mice: Evidence for a role of UCP2 as a regulator of mitochondrial glutathione levels. J. Neurochem..

[78] Zhao B.S., Sun L.K., Jiang X.R., Zhang Y., Kang J.S., Meng H., Li H.Y., Su J. (2019). Genipin protects against cerebral ischemia-reperfusion injury by regulating the UCP2-SIRT3 signaling pathway. Eur J. Pharm..

[79] Della-Morte D., Dave K.R., DeFazio R.A., Bao Y.C., Raval A.P., Perez-Pinzon M.A. (2009). Resveratrol pretreatment protects rat brain from cerebral ischemic damage via a sirtuin 1–uncoupling protein 2 pathway. Neuroscience.

[80] Brandi J., Cecconi D., Cordani M., Torrens-Mas M., Pacchiana R., Dalla Pozza E., Butera G., Manfredi M., Marengo E., Oliver J. (2016). The antioxidant uncoupling protein 2 stimulates hnRNPA2/B1, GLUT1 and PKM2 expression and sensitizes pancreas cancer cells to glycolysis inhibition. Free Radic. Biol. Med..

[81] Cardoso S., Seica R.M., Moreira P.I. (2018). Uncoupling Protein 2 Inhibition Exacerbates Glucose Fluctuation-Mediated Neuronal Effects. Neurotox Res..

[82] Caltagirone C., Cisari C., Schievano C., Di Paola R., Cordaro M., Bruschetta G., Esposito E., Cuzzocrea S., Grp S.S. (2016). Co-ultramicronized Palmitoylethanolamide/Luteolin in the Treatment of Cerebral Ischemia: From Rodent to Man. Transl Stroke Res..

[83] Yeung F., Hoberg J.E., Ramsey C.S., Keller M.D., Jones D.R., Frye R.A., Mayo M.W. (2004). Modulation of NF-kappa B-dependent transcription and cell survival by the SIRT1 deacetylase. Embo J..

[84] Toledo-Pereyra L.H., Toledo A.H., Walsh J., Lopez-Neblina F. (2004). Molecular signaling pathways in ischemia/reperfusion. Exp. Clin. Transplant.: Off. J. Middle East. Soc. Organ. Transplant..

[85] Liu T., Clark R.K., Mcdonnell P.C., Young P.R., White R.F., Barone F.C., Feuerstein G.Z. (1994). Tumor-Necrosis-Factor-Alpha Expression in Ischemic Neurons. Stroke.

[86] Pal G., Vincze C., Renner E., Wappler E.A., Nagy Z., Lovas G., Dobolyi A. (2012). Time Course, Distribution and Cell Types of Induction of Transforming Growth Factor Betas following Middle Cerebral Artery Occlusion in the Rat Brain. PLoS ONE.

[87] Galvin K.A., Oorschot D.E. (2003). Continuous low-dose treatment with brain-derived neurotrophic factor or neurotrophin-3 protects striatal medium spiny neurons from mild neonatal hypoxia/ischemia: A stereological study. Neuroscience.

[88] Ahmad A., Genovese T., Impellizzeri D., Crupi R., Velardi E., Marino A., Esposito E., Cuzzocrea S. (2012). Reduction of ischemic brain injury by administration of palmitoylethanolamide after transient middle cerebral artery occlusion in rats. Brain Res..

[89] Longa E.Z., Weinstein P.R., Carlson S., Cummins R. (1989). Reversible middle cerebral artery occlusion without craniectomy in rats. Stroke; A J. Cereb. Circ..

[90] Melani A., Pantoni L., Corsi C., Bianchi L., Monopoli A., Bertorelli R., Pepeu G., Pedata F. (1999). Striatal outflow of adenosine, excitatory amino acids, gamma-aminobutyric acid, and taurine in awake freely moving rats after middle cerebral artery occlusion: Correlations with neurological deficit and histopathological damage. Stroke; A J. Cereb. Circ..

[91] Guo C., Yin Y., Duan J., Zhu Y., Yan J., Wei G., Guan Y., Wu X., Wang Y., Xi M. (2015). Neuroprotective effect and underlying mechanism of sodium danshensu [3-(3,4-dihydroxyphenyl) lactic acid from Radix and Rhizoma Salviae miltiorrhizae = Danshen] against cerebral ischemia and reperfusion injury in rats. Phytomedicine: Int. J. Phytother. Phytopharm..

[92] Di Paola R., Fusco R., Gugliandolo E., D’Amico R., Campolo M., Latteri S., Carughi A., Mandalari G., Cuzzocrea S. (2018). The Antioxidant Activity of Pistachios Reduces Cardiac Tissue Injury of Acute Ischemia/Reperfusion (I/R) in Diabetic Streptozotocin (STZ)-Induced Hyperglycaemic Rats. Front. Pharm..

[93] Maleki S.N., Aboutaleb N., Souri F. (2018). Berberine confers neuroprotection in coping with focal cerebral ischemia by targeting inflammatory cytokines. J. Chem. Neuroanat..

[94] Ivers N.M., Halperin I.J., Barnsley J., Grimshaw J.M., Shah B.R., Tu K., Upshur R., Zwarenstein M. (2012). Allocation techniques for balance at baseline in cluster randomized trials: A methodological review. Trials.

[95] Petrosino S., Campolo M., Impellizzeri D., Paterniti I., Allarà M., Gugliandolo E., D’Amico R., Siracusa R., Cordaro M., Esposito E. (2017). 2-pentadecyl-2-oxazoline, the oxazoline of pea, modulates carrageenan-induced acute inflammation. Front. Pharmacol..

[96] Schomacher M., Muller H.D., Sommer C., Schwab S., Schabitz W.R. (2008). Endocannabinoids mediate neuroprotection after transient focal cerebral ischemia. Brain Res..

[97] Hara H., Friedlander R.M., Gagliardini V., Ayata C., Fink K., Huang Z., Shimizu-Sasamata M., Yuan J., Moskowitz M.A. (1997). Inhibition of interleukin 1beta converting enzyme family proteases reduces ischemic and excitotoxic neuronal damage. Proc. Natl. Acad. Sci. USA.

[98] Schabitz W.R., Li F., Irie K., Sandage B.W., Locke K.W., Fisher M. (1999). Synergistic effects of a combination of low-dose basic fibroblast growth factor and citicoline after temporary experimental focal ischemia. Stroke A J. Cereb. Circ..

[99] Barber P.A., Hoyte L., Colbourne F., Buchan A.M. (2004). Temperature-regulated model of focal ischemia in the mouse: A study with histopathological and behavioral outcomes. Stroke; A J. Cereb. Circ..

[100] Rodriguez-Sanabria F., Rull A., Beltran-Debon R., Aragones G., Camps J., Mackness B., Mackness M., Joven J. (2010). Tissue distribution and expression of paraoxonases and chemokines in mouse: The ubiquitous and joint localisation suggest a systemic and coordinated role. J. Mol. Histol.

[101] Hernandez-Aguilera A., Sepulveda J., Rodriguez-Gallego E., Guirro M., Garcia-Heredia A., Cabre N., Luciano-Mateo F., Fort-Gallifa I., Martin-Paredero V., Joven J. (2015). Immunohistochemical analysis of paraoxonases and chemokines in arteries of patients with peripheral artery disease. Int. J. Mol. Sci..

[102] Fusco R., D’Amico R., Cordaro M., Gugliandolo E., Siracusa R., Peritore A.F., Crupi R., Impellizzeri D., Cuzzocrea S., di Paola R. (2018). Absence of formyl peptide receptor 1 causes endometriotic lesion regression in a mouse model of surgically-induced endometriosis. Oncotarget.

